# Volatile Organic Compounds From Breath Differ Between Patients With Major Depression and Healthy Controls

**DOI:** 10.3389/fpsyt.2022.819607

**Published:** 2022-07-12

**Authors:** Marian Lueno, Henrik Dobrowolny, Dorothee Gescher, Laila Gbaoui, Gabriele Meyer-Lotz, Christoph Hoeschen, Thomas Frodl

**Affiliations:** ^1^Department of Psychiatry and Psychotherapy, Otto von Guericke University Magdeburg, Magdeburg, Germany; ^2^Institute of Medical Engineering, Otto von Guericke University Magdeburg, Magdeburg, Germany; ^3^Department of Psychiatry, Psychotherapy and Psychosomatics, University Hospital, RWTH Aachen, Aachen, Germany

**Keywords:** depression, biomarker, breath gas, volatile organic compounds, classification, clinical utility

## Abstract

Major depressive disorder (MDD) is a widespread common disorder. Up to now, there are no easy and frequent to use non-invasive biomarkers that could guide the diagnosis and treatment of MDD. The aim of this study was to investigate whether there are different mass concentrations of volatile organic compounds (VOCs) in the exhaled breath between patients with MDD and healthy controls. For this purpose, patients with MDD according to DSM-V and healthy subjects were investigated. VOCs contained in the breath were collected immediately after awakening, after 30 min, and after 60 min in a respective breath sample and measured using PRT-MS (proton-transfer-reaction mass spectrometry). Concentrations of masses m/z 88, 89, and 90 were significantly decreased in patients with MDD compared with healthy controls. Moreover, changes during the time in mass concentrations of m/z 93 and 69 significantly differed between groups. Differentiation between groups was possible with an AUCs of 0.80–0.94 in ROC analyses. In this first study, VOCs differed between patients and controls, and therefore, might be a promising tool for future studies. Altered masses are conceivable with energy metabolism in a variety of biochemical processes and involvement of the brain–gut–lung–microbiome axis.

## Introduction

Mental Health disorders are on the rise in every country in the world. Estimates in Europe are almost €800 billion (US$1 trillion) a year—more than cancer, cardiovascular disease, and diabetes do cost together (1). One of the most common psychiatric diseases is major depressive disorder (MDD) which can effectively be treated with psychotherapy and/or antidepressants. However, still one-third of the patients do not respond to at least two different antidepressant trials and might need it as early as possible with different treatment options (2).

Unfortunately, there are currently no easy and frequently applicable non-invasive biomarkers available that could help to facilitate diagnosis of a depressive episode or that could guide to the most effective therapy. Noteworthy, currently there are ongoing studies on biomarkers in the area of MDD using, e.g., neuroimaging, inflammation markers, hormones, markers for oxidative stress, and genetics. Significant differences were described for MDD compared with healthy controls for functional and structural neuroimaging (3) and as well as analyses of the immune system and inflammation (4). Moreover, the dysregulation of the hypothalamic–pituitary–adrenal (HPA) hormone axis has been well described in MDD (5). In a recent meta-analysis on prospective studies that aimed to predict MDD onset, relapse and recurrence by imaging, genetic information, inflammation markers, and hormone markers, only cortisol was detected to be a significant predictor (6). Thus, signatures related to the cortisol/stress-system seem to be of major relevance. With respect to cortisol tests the cortisol awakening response (CAR) is one of the most straightforward measures of dynamics of the HPA axis and can be obtained reliably (7). The CAR is thought to reflect the sensitivity of the HPA axis to the natural challenge awakening. However, overall, no widely accepted clinical biomarkers for MDD have been developed (8).

The aforementioned measures currently under investigation are invasive blood tests, except the test for cortisol when measured in saliva, which, however, is unpleasant to collect for the participants. It is not convenient for patients to give numerous blood samples throughout the day or even over several days in order to follow the disease course longitudinally. Moreover, functional neuroimaging is expensive and time consuming and it is not feasible to scan patients several times as a routine procedure. A non-invasive longitudinal measurement would certainly increase the information on individual disease characteristics and the dynamics of these during the disease in patients. Thus, new non-invasive markers are highly needed.

One of these possible methods might be breath gas analysis. The exhaled breath gas includes volatile organic compounds (VOCs) that are exhaled shortly after their production, and thus, can deliver information about the acute state of the human being. Therefore, breath biomarkers could provide new insights into metabolic and pathophysiological processes. Breath gas analysis is already clinically implemented, e.g., for alcohol tests. It was found to have predictive power for hyper- or hypoglycemia in human studies (9) and in diet-based studies with mice suffering from obesity (10). Other areas of research with breath gas analysis are pulmonary diseases such as asthma (11) or liver diseases (12).

Currently to our knowledge, no study exists that investigated whether VOCs in breath differ between patients with MDD and healthy controls, although metabolic changes are well known in patients with MDD.

The aim of this study was therefore primarily to identify potential VOCs in breath that differ between patients and controls. Second, we were interested to investigate the change of breath gas markers during the awakening time, since the awakening time was previously under focus of cortisol research in depression. As a third objective, we were interested to examine whether breath gas analysis might have the potential to differentiate between patients and controls and to investigate if multiple measurements over the first hour after awakening might improve classification. Furthermore, we tested for the influence of hospital environment and medication on the detected mass concentrations.

## Materials and Methods

### Participants

From our psychiatric service 26 patients with DSM-V MDD diagnosis and 25 healthy controls were recruited in a prospective design. All the patients were in therapy either as inpatients or outpatients and at time of investigation were mildly to severely depressed. Inclusion criteria for patients were MDD according to DSM-V and age between 18 and 65 years. The latter applied to healthy controls too, who were recruited from the local community matched for age and sex to the patients included into the study. Exclusion criteria for participants were a known alcohol or drug dependency. Neurological disorders and diseases affecting the brain function were further excluded (e.g., hypo- or hyperthyroidism) and also acute internal disorders. Our participants reported no acute gastrointestinal disease. Furthermore, for the patients a current psychotic disorder, depressive episodes with psychotic symptoms, comorbid personality disorder, and suicidal intent were not allowed. Moreover, other psychiatric axis 1 disorders were excluded. For healthy controls any history of a psychiatric disorder was an exclusion criterion. All the patients and controls had to give written informed consent to participate in the study.

### Clinical Assessments

All the subjects were investigated clinically and diagnosis were confirmed using the SCID diagnostic interview (13) by an additional independent investigator. Moreover, they have been examined using standardized questionnaires for psychiatric symptoms: Hamilton Depression Scale (HAMD) (14), Clinical Global Impression (CGI) (15), and Beck Depression Inventory (BDI) (16). The current medication and also the medication during the last 2 weeks before study inclusion were documented for the group of patients receiving antidepressants. Furthermore, the past clinical course was assessed from interviews and case notes within the psychiatric services in particular to stage the recurrence of the disease. Moreover, previous other doctors’ reports were collected. Thus, as exact as possible treatment history for medications and psychotherapy were obtained.

### Breath Gas Analysis

We measured the VOC concentrations as counts per seconds in the breath gas of all the participants (patients as well as controls) with Proton-Transfer-Reaction Mass Spectrometry (PTR-MS). Breath gas probes were taken at awakening, after 30 min, and after 60 min. Awakening was defined when participants got out of the bed in the morning. All the breath gas collection was carried out after awakening in patients and controls on an empty stomach, to avoid contamination with acute food intake or smoking effects. Participants were instructed to sit calm for 2 min and to breath normally and then to start breath gas sampling. Since breath gas was collected at awakening, after 30 min, and after 60 min, tedlar bags were used, which are special tubes for collecting air and breath. No further preparation such as freezing or making use of condensation is necessary. For better detection of the exact substances, we carried out six probes with the PTR-MS system as well as a more sophisticated system based on a time-of-flight (TOF) analyzer unit. The sensitivity and specificity of such a system is by far better than the quadrupole-based system.

Smoking influences some of the VOCs. In the smokers benzene, xylene isomers, styrene, ethylbenzene, acetonitrile, and furan derivatives and also hydrocarbons have been detected in exhaled breath (17). With regards to smoking the patients and test persons behavior was documented and moreover, it can clearly be detected with breath gas analysis and the VOCs concerned were excluded from the analysis.

Therefore, we selected those VOCs that are already known from breath research and are not related to smoking. We determined an effect-size for group differences between patients and controls as *d* = 1.37 for comparison of single values and an effect size of 15 per group. For this first proof of concept study, we intended thus to recruit 25 patients and controls to allow also for covariates to be included in the analyses.

### Data Analysis

Group differences between patients and controls and interactive effects between groups and time (during awakening) were analyzed using general linear models for repeated measures. Correction for multiple comparison was applied using false discovery rate (FDR) and significance was assumed with *p* = 0.05. Cliffs Delta statistics was used to indicate effect sizes for differences between patients and controls irrespective of distribution of values. Effects of antidepressant medication and the clinical environment (inpatients and outpatients) were analyzed further.

Moreover, a random forest analysis for feature extraction was used to cope with the structure of the test set and the measured values. For cross-validation, the data set was divided into ten randomly chosen training and validation data sets in the ratio of 75 and 25%. The algorithm tries to keep the number of patients and healthy subjects in each randomly chosen data set equal. In each validation of the trained random forest model, the importance of each measured value is calculated using the permutation method. After that, calculated importance values were summed for each measured mass. Subsequently, the five masses with the highest summed importance values were then used for logistic regression. Based on the obtained formulas for combination of markers we obtained the predicted values for the model in the test sample. These models were available to our statistician, not to performers or assessors. The obtained results and reference standards were confirmed in the validation sample. A ROC analysis was then carried out to identify sensitivity, specificity, and accuracy for discrimination between patients with MDD and healthy controls.

## Results

There are no significant differences between patients and controls in terms of gender, age, BMI, alcohol consumption and education (Table 1). Per definition, patients with MDD show significant higher depression severity compared to healthy controls. When performing the study, no adverse events did occur.

**TABLE 1:** Description of the sample.

	Control N = 25	MDD N = 26	Sig.
Gender	*f* = 13, *m* = 12	*f* = 16, *m* = 10	Chi = 0.47, *p* = 0.49
Age (years)	34.4 ± 8.2	37.9 ± 12.6	t = 1.2, *p* = 0.24
Education (years)	12.04 ± 3.2	12.15 ± 2.6	t = 0.14, *p* = 0.89
BMI (kg/m^2^)	24.7 ± 4.0	27.3 ± 7.8	t = 1.5, *p* = 0.21
CGI	0.0	3.6, ± 1.4	t = 13.2, *p* < 0.001
BDI-II	1.7, ± 3.8	32.6, ± 10.8	t = 13.5, *p* < 0.001
HDRS	0.12, ± 0.44	17.2, ± 4.9	t = 17.2, *p* < 0.001
Smoking	Yes = 8	Yes = 0	Chi = 9.1, *p* = 0.003
Alcohol	Yes = 5	Yes = 5	Chi = 0.005, *p* = 0.95
Marital status			Chi = 9.3
Single	5	9	*p* = 0.09
Relationship	14	6	
Married	6	6	
Divorced		5	
Medication class	n.a.		n.a.
None		7	
SSRI		7	
SNRI		4	
NASSA		5	
Others		3	

*Depicted are mean values and SDs. SSRI, serotonin reuptake inhibitors; SNRI, serotonin and noradrenaline reuptake inhibitors; NASSA, noradrenaline and selective serotonergic antidepressants (e.g., mirtazapine), others (1 = valdoxane, 1 = nortiptiline, and 1 = bupropione).*

### Group Comparison

**FIGURE 1:**
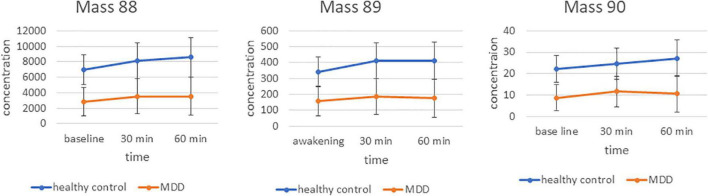
Time course and differences between groups for markers 88, 89, and 90 m/z. Mean concentration in counts per second.

In the analysis of the group and time by group effects, masses were included into repeated measurement ANCOVAs. Interestingly, concentrations of m/z 88 [F(1/50) = 11.2, *p* = 0.0016, *p*_*FDR*_ = 0.0373], m/z 89 [F(1/50) = 10.5, *p* = 0.0022, *p*_*FDR*_ = 0.0373] and m/z 90 [F(1/50) = 11.6, *p* = 0.0013, *p*_*FDR*_ = 0.0373] were significantly decreased in patients with MDD compared with the healthy controls. No significant time effects or time × group interactions were found for these masses (Figure 1). The following masses were altered, but did not survive false discovery rate (FDR) correction: m/z 42 was increased in patients with MDD compared with healthy controls [F(1/50) = 8.3, *p* = 0.006, *p*_*FDR*_ = 0.084]. Decreased in patients with MDD compared with healthy controls were m/z 70 [F(1/50) = 5.3, *p* = 0.025, *p*_*FDR*_ = 0.156), m/z 74 (F(1/50) = 5.6, *p* = 0.022, *p*_*FDR*_ = 0.154], m/z 85 [F(1/50) = 6.3, *p* = 0.016, *p*_*FDR*_ = 0.128], m/z 91 [F(1/50) = 7, *p* = 0.011, *p*_*FDR*_ = 0.121], and m/z 93 [F(1/50) = 6.6, *p* = 0.013, *p*_*FDR*_ = 0.121].

Moreover, different time courses during the awakening for the concentrations of the m/z 93 [F(1/50) = 5.2, *p* = 0.007 with assumed sphericity] and 69 [F(1/50) = 2.6, *p* = 0.091 with Greenhouse–Geisser correction] were detected between patients with MDD and controls (Supplementary Figure 1). The difference between m/z 93 at 30 min minus baseline was significantly higher for patients with MDD compared with the controls [F(1/50 = 13.5, *p* = 0.00074, *p*_*FDR*_ = 0.011]. The difference between m/z 69 at 60 min minus 30 min is significantly different between groups as well [F(1/50) = 4.6, *p* = 0.037, *p*_*FDR*_ = 0.041].

### Candidate Selection and Evaluation Using Random Forest Analysis

**FIGURE 2:**
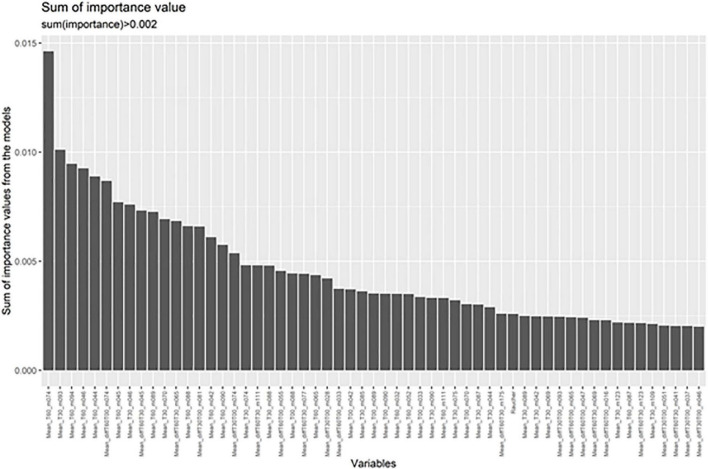
Random forest analyses indicate the masses most important for differentiating between patients and controls. These are shown in order of importance.

In order to test whether markers not showing significant differences in the multiple testing corrected ANCOVA might contribute to differentiation between groups a random forest analysis was automatically run on those masses showing uncorrected significant differences (Supplementary Tables 1, 2). The random forest analysis was run for each effect size threshold ten times with varying training and validation samples that were automatically chosen by the software. Taking breath gas markers from baseline measurements at awakening resulted in good accuracy of 80%. This was improved by adding measurements from the 30 and 60 min time points as well. Moreover, including the differences between measurement points (increase, decrease over time) further improved the accuracy. From the random forest analysis most important markers for the models are derived (Figure 2).

### Composite Marker and Classification Analysis Based on Logistic Regression

**FIGURE 3:**
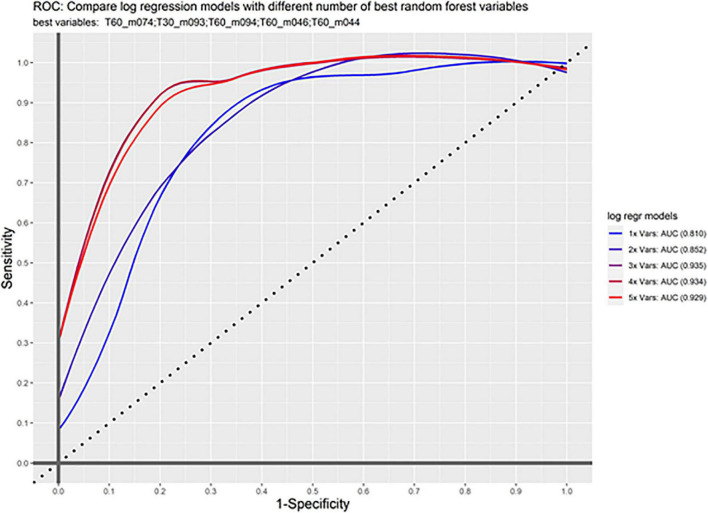
ROC curve. Shown is sensitivity and 1-specificity for the combination of markers in the validation sample. First m/z 74 was calculated alone (1x Vars), then subsequently the other important markers are added showing an increase up to the first three markers.

Based on the selected markers from the random forest analysis, we further investigated the classification accuracy in our test and the validation samples. Prediction accuracy was already high for the most important VOC (m/z 74) measured 60 min after awakening even in the validation sample (AUC 0.81). Taking the three best markers from the random forest analysis (m/z 74 at 60 min, m/z 94 at 60 min, and m/z 93 at 30 min) showed a very good accuracy in the validation sample (AUC 0.93). Adding the next markers to the model does not further improve the classification (Figure 3).

Adding the marker m/z 69 showed very good classification. The classification was as following: AUC = 0.96 in the test sample and AUC = 0.91 in the validation sample (Supplementary Figure 2).

### Analysis of Influence From the Environment and Medication

Moreover, we tested whether breath gas sampling in the hospital for inpatients differed from breath gas sampling at home considering depression severity, age, and gender as covariates. Therefore, the six patients being in outpatient therapy were compared with the 19 patients in the hospital. No significant difference was found in the aforementioned significant different breath gas markers, except m/z 74. Levels of m/z 74 were significantly lower in patients sampling at home compared to those sampling in the hospital [F(1/24) = 6.2, *p* = 0.02]. No significant difference in concentration of these VOCs was detected between patients on antidepressants compared with those without antidepressants.

## Discussion

Endogenous VOCs provide a plethora of information regarding the metabolic processes. This is to our knowledge the first study using breath gas analysis to explore potential signatures for supporting diagnostics in MDD.

Some of the mass signatures found to be associated with MDD have a well-known origin. Mass signatures that were found to be significantly and stably reduced over the awakening period in patients compared with controls can be traced back butanoic or butyric acid (89 m/z) (18). Butyric acid plays an important role in oxidative generation of ATP, supplies anabolic pathways (gluconeogenesis and lipogenesis) and contributes to the regulation of cell metabolism by triggering signaling pathways (19). It is produced in the colon through microbial fermentation of dietary fiber that is primarily absorbed by colonocytes and metabolized in the liver for the generation of ATP during energy metabolism. Some butyric acid is absorbed in the distal colon, which is not connected to the portal vein, thereby allowing for the systemic distribution of butyric acid to multiple organ systems through the circulatory system (19). Reduction of butyric acid is in line with new findings from a large microbiome study that shows that patients with MDD have reduced amounts of butyric acid producing bacteria in the gut that in turn are associated with reduced quality of life (20). Moreover, in one study, using 16S rRNA gene-based next generation sequencing a total of 40 bacterial taxa were found to be altered in saliva of patients with MDD compared with healthy controls (21). Thus, oral microbiome and not only gut or lung microbiome might need to be considered when locating origins of altered VOCs.

It always is the question if an association like the one found in the present study is causal or not. Patients with MDD reduce their physical activity and thus the changes found here might be associated with the physical activity. In a study on prolonged exercise in not depressed subjects short-chain fatty acids (SCFAs) represented by acetic acid, butanoic acid, and propionic acid increased (22). In an animal experiment, it could be shown that rats that voluntarily exercise have an increased level of butyrate in their intestines (23). Butyrate is an ester of butyric acid. This would argue that reduced physical activity might be a contributing factor to butyric acid in breath. Thus, further studies are necessary to study the effect on exercise butyric acid in patients with MDD.

One other marker with relevance to MDD was isoprene (69 m/z) (24), which is derived from the cholesterol synthetic pathway (25). Cholesterol blood levels are significantly increased in patients with MDD compared with the healthy controls and are positively associated with depression severity (26). Isoprene was found to be reduced in breath gas in patients with MDD in particular at awakening compared with healthy controls in our study. During the awakening period the levels of the marker in controls approaches that of patients with MDD. Since isoprene is stored in muscles and gets released during muscle contraction (27), our finding might suggest that reduced activity in patients with MDD is associated with reduced isoprene levels despite increase of cholesterol levels. However, isoprene was found to decrease during prolonged exercise in humans (22). In acute exercise, this might be different due to pulmonary ventilation, where increases of isoprene have been detected. Moreover, m/z 74, m/z 93, and m/z 94 were not found to be associated with exercise so far, we are aware of in other studies.

It could also be proven for isoprene that it can be measured in increased concentration in the air breathed during physical activity. Here, it could be proven that the measured concentration also depends on the lung blood flow. Endogenous production is not increased during increased activity (28).

M/z 93 was significantly increased in MDD compared with the controls and significantly increased during the awakening response in patients more than in the healthy controls. This mass belongs to toluene (29), which is predominantly used as an industrial feedstock. Its exposure was previously found to be associated with cognitive dysfunctioning in the neuropsychological tests (30).

Currently, diagnostics are still based on clinical interviews, psychiatric history and assessment of psychopathology. Breath gas analysis has the advantage against blood analyses or functional MRI that it is a non-invasive, easy to apply measurement. Measurement in patients with MDD was well-tolerated and is feasible also over several time points during the day. Thus, it allows multiple measurements over time, e.g., during the awakening period like in our study. In our experience, subjects were comfortable with the procedure even when they were severely depressed and were not stressed by it. Breath gas analysis did show a very good accuracy in predicting patients with MDD vs. healthy subjects in the test as well as the validation sample. An AUC of 0.93 for a three-factor solution seems to be promising for the next step in studying these signatures in the larger clinical studies including also other diagnoses.

The three most important markers from the random forest analysis were markers 74, 93, and 94. While m/z 93 is toluene (29), the origin of m/z 94 is unclear. For marker 74, lower levels were found in patients compared with the healthy controls. Interestingly, patients sampling at home showed the lowest levels suggesting that this marker might differ even more, when investigated only in the usual home environment. One possibility for the significantly higher levels in hospitalized patients could be that patients in the hospital receive more intense therapy, a professionally chosen diet plan and nursing support to motivate patients with depression to take their meals despite low appetite. Using TOF-PTR-MS analysis with a 100-fold better detection in addition to PTR–MS, we tentatively relate this mass to protonated methylguanidine (74 m/z), which is a guanidine compound and can be synthesized from creatine and creatinine (31). It might reflect creatine metabolism, but it also was found to be highly dependent on the diet, e.g., from boiled beef and fish (32). Therefore, the difference between in and outpatients might be due to better nutrition in the hospital. Interestingly, creatine deficiency was associated with MDD and in turn its supplementation showed positive therapeutic effects (33).

In this first study, the sample size was small thus limiting the generalizability of the findings. In a follow-up study, the sample size needs to be extended toward more outpatients treated in the community and patients in remission as well as longitudinal assessments during the disease course. This will allow to explore whether the markers are trait or state dependent. While in the present study, antidepressant medication seems not to have a major effect, it should then be tested whether this might be the case for all the antidepressant classes. The type of diet can have an impact on metabolites excreted, and thus, these effects can be seen in exhaled breath. In this first attempt at a study, the exact components of the diet were not explicitly asked about. Thus, nutrition needs to be taken into consideration. So far, we are not aware of any of the masses we found to be associated with nutrition, but given the limited number of existing studies, and the small sample in our pilot study, it seems to be important to control for nutrition. Thus, breath gas analysis was carried out in patients and controls at awakening and the hour afterward on an empty stomach. In our sample, patients were more often smokers than non-smokers. Although VOCs known to be associated with smoking were excluded from analyses, in a larger sample the effect of smoking needs to be investigated.

In summary, the present study shows that breath gas analysis might be an interesting method to investigate the metabolic profile in patients with major depressive disorder (MDD).

## Data Availability Statement

The raw data supporting the conclusions of this article will be made available by the authors, without undue reservation.

## Ethics Statement

The studies involving human participants were reviewed and approved by Ethics Committee of medical faculty of Otto von Guericke University Magdeburg. The patients/participants provided their written informed consent to participate in this study.

## Author Contributions

ML: acquisition, analysis and interpretation of the data, and drafting the manuscript. HD: analysis and interpretation of the data and revising the manuscript. LG and DG: interpretation of the data and revising the manuscript. GM-L: acquisition, interpretation of the data, and revising the manuscript. CH: design of the work, interpretation of the data, and revising the manuscript. TF: design of the work, acquisition, analysis and interpretation of the data, and drafting the manuscript. All authors agreed to be accountable for all aspects of the work ensuring that questions related to the accuracy or integrity of any part of the work are appropriately investigated, resolved, and gave approval for the final version.

## Conflict of Interest

The authors obtained a patent for breath gas analysis in major depressive disorder. Thus, there is no embargo for the publication any more. TF received fees for presentation from Janssen-Cilag, Servier, Neuraxpharm, Recordati, and Otsuka. The remaining authors declare that the research was conducted in the absence of any commercial or financial relationships that could be construed as a potential conflict of interest.

## Publisher’s Note

All claims expressed in this article are solely those of the authors and do not necessarily represent those of their affiliated organizations, or those of the publisher, the editors and the reviewers. Any product that may be evaluated in this article, or claim that may be made by its manufacturer, is not guaranteed or endorsed by the publisher.
