# Supplementary material for: Volatile Organic Compounds From Breath Differ Between Patients With Major Depression and Healthy Controls
Source: Front Psychiatry. 2022 Jul 12;13:819607. doi: 10.3389/fpsyt.2022.819607 (PMC9314777; doi:10.3389/fpsyt.2022.819607)
Supplement: Supplementary file 1 [file Data_Sheet_1.pdf]

Title:

Personalized Breathomics in Major Depression to support Diagnostics

Lueno M, Dobrowolny H, Gaboui L, Meyer-Lotz G, Gescher D, Hoeschen C, Frodl T

Correspondence to
T. Frodl
Department of Psychiatry, Psychotherapy and Psychosomatics
Universitätsklinikum Aachen, RWTH University Aachen
Pauwelsstr. 30
52074 Aachen
Germany
Tel.: 0049-2418089632
Email: tfrodl@ukaachen.de

Supplemental Figure 1: Time by group interaction shown for markers 69 and 93.

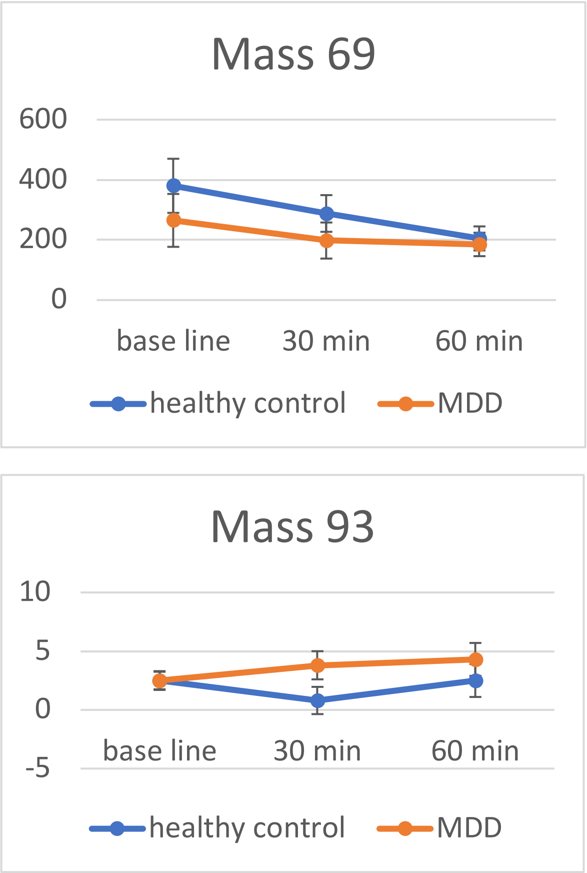

Supplemental Table 1 Cliffs Delta statistics to obtain effect sizes for breath gas signatures significantly different between patients and controls.

	Sample	Name	value
1	Test	Mean_T60_m074	-0.6225
2	Test	Mean_T60_m056	-0.575
3	Test	Mean_T60_m094	-0.53
4	Test	Mean_T30_m046	-0.5225
5	Test	Mean_T30_m074	-0.52
6	Test	Mean_T60_m046	-0.505
7	Test	Mean_diffT30T00_m081	0.5
8	Test	Mean_T30_m085	-0.4825
9	Test	Mean_T30_m093	0.475
10	Test	Mean_T60_m042	0.46
11	Test	Mean_diffT60T30_m077	0.4525
12	Test	Mean_diffT60T00_m065	0.445
13	Test	Mean_T00_m090	-0.445
14	Test	Mean_diffT30T00_m093	0.4425
15	Test	Mean_T60_m032	-0.44
16	Test	Mean_diffT60T30_m045	0.43
17	Test	Mean_T30_m044	-0.4275
18	Test	Mean_T60_m065	0.42
19	Test	Mean_T00_m042	0.42
20	Test	Mean_T00_m070	-0.4175
21	Test	Mean_T30_m087	-0.4175
22	Test	Mean_diffT60T30_m100	0.4175
23	Test	Mean_diffT60T30_m065	0.4125
24	Test	Mean_T00_m085	-0.41
25	Test	Mean_T60_m037	0.41
26	Test	Mean_diffT30T00_m040	0.395
27	Test	Mean_T00_m089	-0.395
28	Test	Mean_T30_m060	-0.3925
29	Test	Mean_T60_m045	0.39
30	Test	Mean_diffT60T30_m123	0.39
31	Test	Mean_diffT60T30_m175	-0.39
32	Test	Mean_T30_m109	-0.385
33	Test	Mean_T60_m044	-0.385
34	Test	Mean_T30_m069	-0.385

Supplemental Table 2 Mass differences between patients and controls: Shown are significant different masses (uncorrected)

**Mass**	**Patients with MDD**	**Healty Controls**	**F (1/50)**	**p-value**	**pFDR corrected**
42 bl	111.1 ±143.8	30.7 ±36	7.6	0.00847	0.09317
42 30min	114.2 ±166	24.2 ±16.5	7.6	0.00841	0.09317
42 60min	120.5 ±180.8	17.7 ±11.9	8.4	0.00565	0.09317
44 60min	42.7 ±31.9	83.8 ±61.3	9.8	0.003	0.09317
46 30min	169.8 ±158.5	805 ±1457.4	4.2	0.04608	0.26218
46 60min	144.3 ±142.9	367.9 ±408.4	5.7	0.02098	0.18274
52 60min	1.4 ±1.6	2.9 ±3.2	4.7	0.03526	0.23272
69 30min	197.8 ±124.7	288 ±170.5	4.5	0.03926	0.24915
70 30min	11.3 ±8.7	17.5 ±10.3	4.3	0.0447	0.26218
74 30min	18.8 ±13.2	35 ±30.7	4.9	0.03146	0.21629
74 60min	17.9 ±11.5	32.6 ±12.3	18	0.0001	0.0165
79 30min	5.5 ±6.3	2.2 ±2.1	4.9	0.0311	0.21629
85 bl	6.2 ±5	10 ±5.8	5.6	0.02215	0.18274
85 30min	5.8 ±6.2	9.8 ±5.9	5.6	0.02205	0.18274
87 60min	15 ±11.1	25.3 ±22.2	5	0.02949	0.21629
88 bl	2908.3 ±3260.7	6887.4 ±5864.5	9.2	0.0031	0.09317
88 30min	3669.6 ±4038.8	8010.5 ±7012.3	8.3	0.00607	0.09317
88 60min	3686.6 ±4261.5	8453 ±7810.3	8	0.00692	0.09317
89 bl	162.6 ±178.1	336.6 ±273.9	8.1	0.00631	0.09317
89 30 min	192.5 ±198.2	403.8 ±356.2	7.8	0.00739	0.09317
89 60min	181.8 ±199.1	404.9 ±371	7.8	0.00743	0.09317
90 bl	9.1 ±8.9	21.9 ±19.8	9.6	0.00323	0.09317
90 30min	12 ±13.8	24.2 ±22.1	6.3	0.01539	0.15685
90 60min	11 ±12.6	26.6 ±27.2	7.6	0.00796	0.09317
91 30min	4.3 ±2.6	6.6 ±3.9	5.3	0.0262	0.20586
93 30min	3.9 ±3.9	0.8 ±1.6	12.8	0.0082	0.06765
94 60min	4.2 ±2.9	11.5 ±12.7	7.7	0.00776	0.09317
